# P190B RhoGAP Regulates Chromosome Segregation in Cancer Cells

**DOI:** 10.3390/cancers4020475

**Published:** 2012-04-25

**Authors:** Melissa Hwang, Sirisha Peddibhotla, Peter McHenry, Peggy Chang, Zachary Yochum, Ko Un Park, James Cooper Sears, Tracy Vargo-Gogola

**Affiliations:** 1 Department of Biochemistry and Molecular Biology and the Indiana University Simon Cancer Center, Indiana University School of Medicine, 1234 Notre Dame Avenue, South Bend, IN 46617, USA; E-Mails: melissa.e.hwang@gmail.com (M.H.); pchang2@alumni.nd.edu (P.C.); zachary.a.yochum.1@nd.edu (Z.Y); kounpark@iupui.edu (K.U.P.); jsears22@gmail.com (J.C.S); 2 Department of Molecular and Human Genetics, Baylor College of Medicine, John P. McGovern Campus, NABS-0250, Houston, TX 77030, USA; E-Mail: sirishap@bcm.edu; 3 Department of Biology, Southwestern Adventist University, 100 W. Hillcrest, Keene, TX 76059, USA; E-Mail: pmchenry@swau.edu

**Keywords:** p190B RhoGAP, mitosis, chromosome segregation, centrosome, aneuploidy, Rho GTPases, breast cancer

## Abstract

Rho GTPases are overexpressed and hyperactivated in many cancers, including breast cancer. Rho proteins, as well as their regulators and effectors, have been implicated in mitosis, and their altered expression promotes mitotic defects and aneuploidy. Previously, we demonstrated that p190B Rho GTPase activating protein (RhoGAP) deficiency inhibits ErbB2-induced mammary tumor formation in mice. Here we describe a novel role for p190B as a regulator of mitosis. We found that p190B localized to centrosomes during interphase and mitosis, and that it is differentially phosphorylated during mitosis. Knockdown of p190B expression in MCF-7 and Hela cells increased the incidence of aberrant microtubule-kinetochore attachments at metaphase, lagging chromosomes at anaphase, and micronucleation, all of which are indicative of aneuploidy. Cell cycle analysis of p190B deficient MCF-7 cells revealed a significant increase in apoptotic cells with a concomitant decrease in cells in G1 and S phase, suggesting that p190B deficient cells die at the G1 to S transition. Chemical inhibition of the Rac GTPase during mitosis reduced the incidence of lagging chromosomes in p190B knockdown cells to levels detected in control cells, suggesting that aberrant Rac activity in the absence of p190B promotes chromosome segregation defects. Taken together, these data suggest that p190B regulates chromosome segregation and apoptosis in cancer cells. We propose that disruption of mitosis may be one mechanism by which p190B deficiency inhibits tumorigenesis.

## 1. Introduction

The Rho signaling network that includes Rho GTPases and their regulators and effectors regulates a diverse set of cellular functions such as cell cycle progression, mitosis, migration, apoptosis, morphogenesis, and cytoskeletal organization [1]. Rho GTPases are overexpressed and hyperactivated in several types of cancer, including breast cancer [2,3], and aberrant Rho signaling has been implicated in all stages of cancer development and progression (for review see [4]).

Aneuploidy and genomic instability are features of most solid tumors [5], and disruption of mitosis and cytokinesis facilitates these processes. A number of studies have implicated Rho GTPases and their regulators and effectors in centrosome duplication, mitotic spindle formation, kinetochore-microtubule attachments, and cytokinesis [6,7,8,9,10]. Perturbation of any of these processes can lead to abnormal chromosome segregation and aneuploidy.

Rho GTPases cycle between active GTP-bound and inactive GDP-bound states, and tight regulation of Rho GTPase activity is important for proper control of a diverse set of cellular functions [11]. The Rho GTPase cycle is positively regulated by guanine nucleotide exchange factors (GEFs) and negatively regulated by the Rho GTPase activating proteins (RhoGAPs) and guanine nucleotide dissociation inhibitors (GDIs). Altered expression of Rho regulators has been shown to affect mammary tumor formation in mice [12,13,14]. Previous studies from our laboratory demonstrated that heterozygous expression of p190B, a major regulator of Rac and RhoA [15,16], potently inhibited tumor formation and metastases in the MMTV-Neu mouse mammary tumor model [17]. Conversely, ectopic expression of p190B in the mammary epithelium of MMTV-Neu mice increased both tumor burden and metastasis [18]. The objective of the current study was to identify potential mechanisms by which p190B influences tumorigenesis. Because aberrant Rho signaling has been implicated in several stages of mitosis and cytokinesis and the closely related p190A RhoGAP has been shown to be an important regulator of cytokinesis [19,20,21,22], we hypothesized that altered p190B expression may also disrupt mitotic processes. Here we show that p190B affects chromosome segregation in MCF-7 breast cancer cells and Hela cervical cancer cells.

## 2. Results

### 2.1. P190B RhoGAP Localizes to Centrosomes and Is Phosphorylated During Mitosis

**Figure 1:**
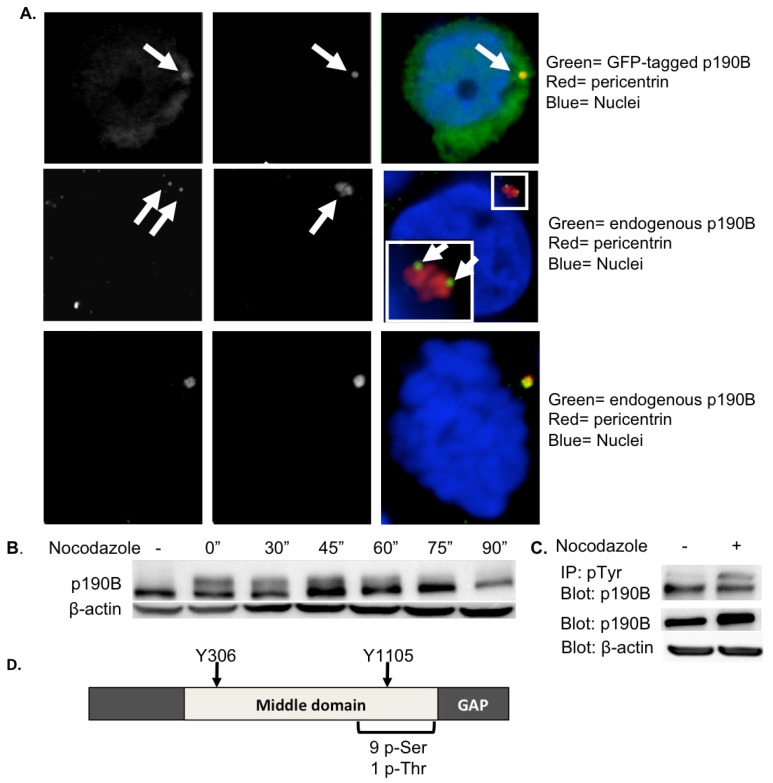
P190B RhoGAP localizes to centrosomes and is phosphorylated during mitosis. Confocal images are shown demonstrating co-localization of GFP-tagged p190B and endogenous p190B protein with pericentrin in interphase MCF-7 cells, as well as co-localization of endogenous p190B with pericentrin in a mitotic MCF-7 cell (**A**). Western blot of nocodazole synchronized MCF-7 cells reveals a slower migrating form of p190B during mitosis. β-Actin is shown as a loading control (**B**). Western blot of immunoprecipitated pTyr proteins from asynchronous and synchronized MCF-7 cell lysates probed with an anti-p190B antibody demonstrating that p190B is differentially phosphorylated on Tyr residues during mitosis (**C**). P190B levels are comparable in asynchronous and synchronized lysates as shown by a Western blot loaded with 10% input and probed with p190B antibody. β-Actin is shown as a loading control. Diagram depicts p190B phosphorylation sites (**D**). P190B is phosphorylated on 9 Ser residues and 1 Thr residue during mitosis.

To begin to elucidate the function of p190B, we first investigated its cellular localization by immunofluorescence staining. In MCF-7 breast cancer cells, we detected GFP-tagged p190B and endogenous p190B at the centrosomes during interphase, as well as endogenous p190B at the centrosomes during mitosis (Figure 1A). To determine whether p190B expression levels are regulated during mitosis, we measured protein levels throughout mitosis by Western blot in nocodazole-synchronized MCF-7 cells. No significant changes in p190B protein levels were detectable in mitotic cells from three independent experiments (data not shown). However, a slower-migrating p190B band was apparent in mitotic cell lysates that is consistent with protein phosphorylation (Figure 1B), and p190B is phosphorylated at both Ser/Thr and Tyr residues [23,24]. A proteomic study of mitotic phosphoproteins showed that p190B is selectively phosphorylated during mitosis on one Thr and nine Ser residues [25]. The Ser/Thr residues are present at the C-terminal portion of the middle domain and flank Tyr1105 (Figure 1D), which corresponds to a Src kinase consensus site found in p190A [26,27]. To determine whether p190B is also selectively tyrosine phosphorylated during mitosis, we performed immunoprecipitation of phospho-Tyr proteins in lysates prepared from asynchronous and nocodazole-treated mitotic cell populations and probed for p190B on a Western blot. A single band of approximately 190 kDa was observed in the asynchronous and mitotic lysates, whereas an additional slower migrating band was observed in the mitotic cell lysate (Figure 1C). These data suggest that p190B is phosphorylated on tyrosine residues throughout the cell cycle and that it is differentially phosphorylated during mitosis. These results together with published studies [25] indicate that p190B localizes to centrosomes and that it is differentially phosphorylated during mitosis.

### 2.2. P190B Deficiency Enhances Chromosome Segregation Defects in MCF-7 and Hela Cells

**Figure 2:**
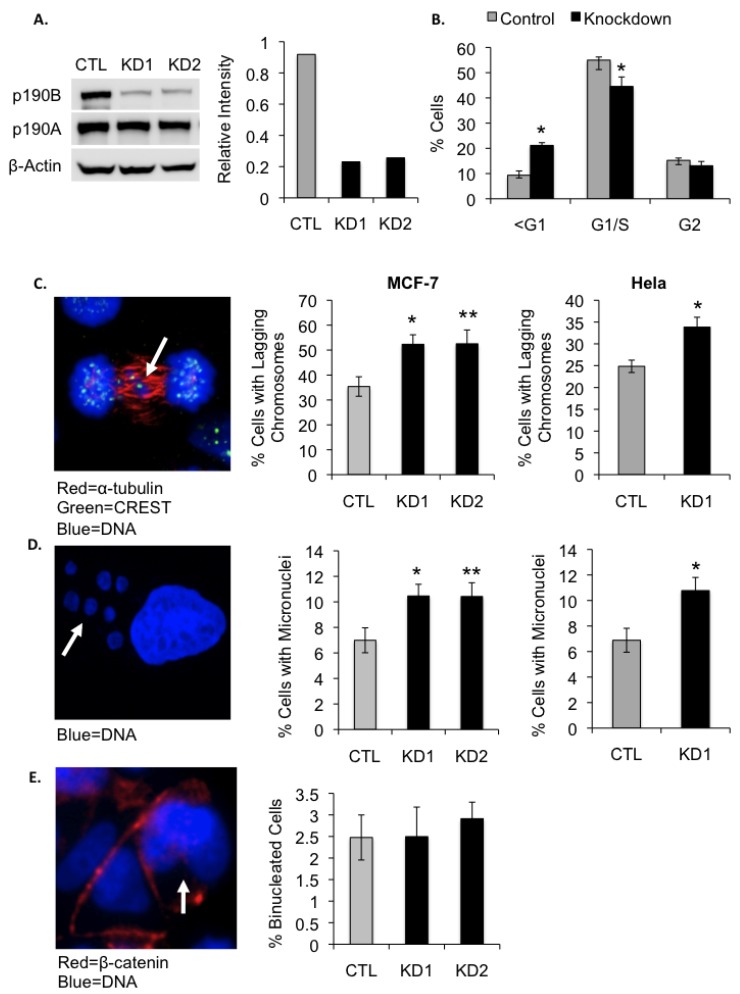
P190B deficiency induces lagging chromosomes and micronucleation in MCF-7 and Hela cells. Western blot and graph representing normalized densitometry values show an approximately 75% reduction in p190B protein levels in MCF-7 cells transfected with p190B-targeting siRNA compared to cells transfected with control non-targeting siRNA. siRNA targeting P190B did not affect the expression levels of the closely related p190A RhoGAP as determined by Western blotting (**A**). The percentages of control and p190B knockdown cells in different stages of the cell cycle as determined by flow cytometry are graphed, * *p* < 0.001 (**B**). A representative confocal image of an anaphase MCF-7 cell immunostained with an antibody against α-tubulin and CREST anti-serum is shown. Arrow indicates a lagging chromosome. The percentages of control and p190B deficient MCF-7 and Hela cells with lagging chromosomes at anaphase are graphed, * *p* = 0.012, ** *p* = 0.03 (**C**). A representative confocal image of an interphase MCF-7 cell stained with DAPI is shown. Arrow indicates micronuclei. The percentages of control and p190B knockdown MCF-7 and Hela cells with micronuclei are graphed. For MCF-7 * *p* = 0.027, ** *p* = 0.04 and for Hela, * *p* = 0.019 (**D**). A representative image of MCF-7 cells immunostained with an antibody against β-catenin is shown. DAPI was used to stain DNA. Arrow indicates a binucleated cell. The percentages of binucleated control and p190B deficient MCF-7 cells are graphed (**E**).

To better understand the functional role of p190B in mitosis, we used RNA interference to knockdown p190B protein expression in both MCF-7 and Hela cells. MCF-7 cells transfected with siRNA directed against p190B (labeled KD1 and KD2) exhibited an approximately 75% reduction in p190B protein levels compared to cells transfected with a control (CTL) non-targeting siRNA (Figure 2A). Hela cells transfected with KD1 exhibited similar levels of protein knockdown (data not shown). Neither of the siRNAs targeting p190B affected the expression levels of the closely related p190A protein as determined by Western blot (Figure 2A). To determine how knockdown of p190B affects cell cycle progression, we examined the cell cycle profile of MCF-7 cells transfected with non-targeting and p190B-targeting siRNA (Figure 2B). Cells were fixed and stained with propidium iodide 48 h after transfection and analyzed by flow cytometry. We observed a significant increase in the apoptotic, sub-G1, population in knockdown cells compared to controls (21.2% *vs*. 9.3%, *p* < 0.001), and a concomitant decrease in the G1 and S populations (44.0% *vs*. 54.7% combined G1/S population, *p* < 0.001). These data suggest that loss of p190B leads to cell death at the G1/S transition.

Next we wanted to determine whether loss of p190B caused mitotic defects in the cells that successfully entered mitosis. For this, we quantified lagging chromosomes at anaphase in nocodazole-synchronized MCF-7 and Hela cells transfected with control non-targeting or p190B-targeting siRNA. Lagging chromosomes are indicative of mitotic spindle abnormalities and are a known cause of aneuploidy [28]. Interestingly, p190B deficiency resulted in a significant increase in the number of cells exhibiting lagging chromosomes at anaphase (52.3% and 52.5% in KD1 and KD2 *vs*. 35.4% in control, *p* = 0.012 and 0.030, respectively, for MCF-7; 33.8% in KD1 *vs*. 24.8% in control, *p* = 0.012 for Hela) (Figure 2C). In order to determine whether the observed lagging chromosomes resulted in aneuploidy in our cells, we quantified micronuclei, which are indicative of extra genetic material that can result from improper chromosome segregation [29]. P190B deficiency also resulted in a significant increase in the percentage of MCF-7 and Hela cells containing micronuclei at interphase (10.5% and 10.4% in KD1 and KD2 *vs*. 6.99% in control, *p* = 0.027 and 0.040, respectively, for MCF-7; 9.4% in KD1 *vs*. 6.9% in control, *p* = 0.019 for Hela) (Figure 2D). Because the related p190A RhoGAP plays an important role in cytokinesis [19,20,21,22], we also asked whether p190B deficiency in MCF-7 cells affected the incidence of multinucleated cells, which are indicative of failed cytokinesis. We stained cells with an antibody against E-cadherin to clearly delineate individual cells and quantified the percentage of cells with multiple nuclei. As shown in Figure 2E, p190B deficiency did not affect the rate of multinucleation in MCF-7 cells (2.5% and 2.9% in KD1 and KD2 *vs*. 2.5% in control, *p* = 0.98 and *p* = 0.53, respectively). Together these data indicate that p190B loss in cancer cells increases abnormal chromosome segregation during anaphase, but that its function is dispensable for cytokinesis.

### 2.3. P190B Deficiency Increases the Incidence of Abnormal Microtubule-Kinetochore Attachments

**Figure 3:**
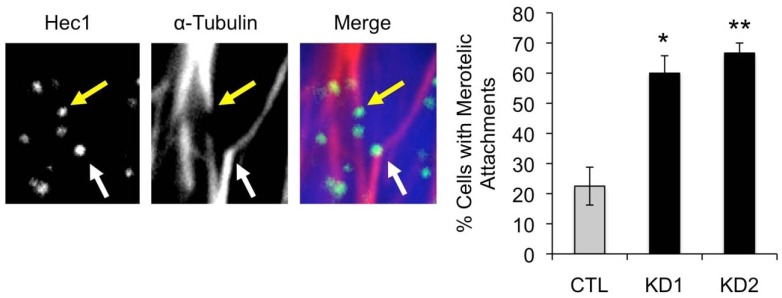
P190B deficiency increases the incidence of merotelic microtubule-kinetochore attachments. Representative confocal images of metaphase MCF-7 cells stained with antibodies against Hec1 and α-tubulin are shown. Yellow arrow indicates a normal attachment and white arrow indicates a merotelic attachment. The percentages of control and p190B deficient MCF-7 cells with merotelic attachments are graphed, * *p* = 0.008 and ** *p* = 0.003.

The major cause of lagging chromosomes at anaphase is the phenomenon of merotelic attachment, where microtubules emanating from both spindle poles attach to the same kinetochore [30]. This frequently results in missegregation as the chromosome remains suspended between the two poles until one microtubule exerts a stronger pull. We therefore quantified merotelic attachments in metaphase MCF-7 cells transfected with control or p190B-targeting siRNA using high-resolution confocal microscopy. We observed that p190B deficiency resulted in a significant increase in the number of cells containing merotelic attachments compared to control cells (60.0% and 66.7% in KD1 and KD1 *vs*. 22.5% in control, *p* = 0.008 and 0.003, respectively) (Figure 3), suggesting that loss of p190B perturbs microtubule-kinetochore attachments. The persistence of this defect into anaphase may reflect a greater frequency of microtubule-kinetochore misattachment in the p190B deficient cells or failure of the cells to correct the misattachments.

### 2.4. Localization of the Chromosomal Passenger Complex Proteins Survivin and Aurora B Is Not Altered in p190B Deficient Cells

**Figure 4:**
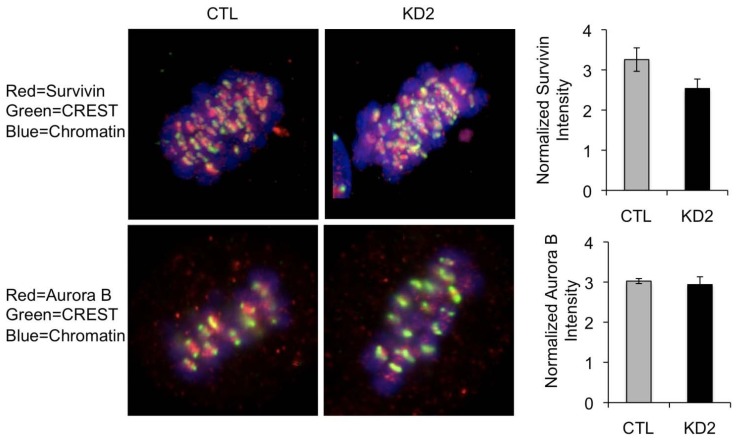
Localization of the CPC proteins Survivin and Aurora B at kinetochores in metaphase MCF-7 cells is not affected by p190B deficiency. Representative confocal images of metaphase control and p190B deficient MCF-7 cells immunostained with antibodies to detect Survivin, Aurora B, or CREST anti-serum are shown. Fluorescence intensities of Survivin and Aurora B normalized to CREST are graphed. Data is representative of at least 30 cells per group quantified from 3 independent experiments.

In order to untangle the mechanisms by which p190B deficiency leads to defects in chromosome segregation, we examined the levels of Survivin and Aurora B, two members of the chromosomal passenger complex (CPC), which is known to play an important role in correcting merotelic attachments [31]. We quantified the fluorescence intensity in metaphase MCF-7 cells stained for Survivin and Aurora B. Fluorescence intensity for each cell was normalized to the intensity of the kinetochore marker CREST. We found a modest decrease in the intensity of Survivin (2.5 in KD1 *vs*. 3.3 in control, *p* = 0.018), and no significant difference in the intensity Aurora B (3.9 in KD1 *vs*. 3.0 in control, *p* = 0.72) (Figure 4), suggesting that the CPC is not differentially activated in the p190B deficient cells compared with controls.

### 2.5. Inhibition of Rac Reduces Chromosome Segregation Defects in p190B Deficient Cells

**Figure 5:**
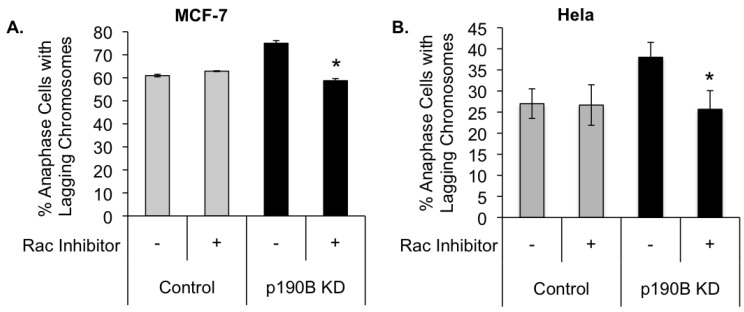
Inhibition of Rac activity reduces the incidence of lagging chromosomes in p190B deficient MCF-7 (A) and Hela (B) cells to control levels. Control and p190B deficient cells were synchronized with nocodazole and fresh media with or without NSC 23766 was added at the time of nocodazole release. The percentages of cells with lagging chromosomes at anaphase are graphed, *p* = 0.0004 for MCF-7 and *p* = 0.02 for Hela.

Rho GTPases, including Cdc42, RhoA, and Rac1, are important regulators of microtubule polymerization and stability in migrating cells [32]. However, the contribution of Rho GTPases to microtubule function during mitosis is not well understood. P190B is a known inhibitor of Rac and RhoA [15,16], and our previous studies have implicated p190B as an important regulator of Rac activity during mammary tumorigenesis [17]. We therefore hypothesized that elevated Rac activity may perturb microtubule dynamics to increase the incidence of lagging chromosomes in the p190B deficient cells. To test this, MCF-7 cells transfected with control or p190B-targeting siRNA were arrested with nocodazole and treated with the Rac inhibitor NSC 23766 at the time of release from nocodazole. We then quantified the percentage of anaphase cells with lagging chromosomes and found that treatment with the Rac inhibitor restored the percentage of lagging chromosomes to control levels (58.7% lagging chromosomes in KD1 with Rac inhibitor *vs*. 75.0% in KD1 without inhibitor, *p* = 0.0004) (Figure 5A). This experiment was repeated in Hela cells with similar results (38.0% lagging chromosomes in KD1 with Rac inhibitor *vs*. 25.7% in KD1 without inhibitor, *p* = 0.02) (Figure 5B). These data suggest that elevated Rac activity in p190B deficient cells is responsible, at least in part, for the increase in chromosome segregation defects detected in p190B knockdown cells.

## 3. Discussion

P190B and p190A RhoGAP have been shown to have both overlapping and distinct functions in central nervous system and mammary gland development [33,34,35]. Although p190A has previously been shown to regulate cytokinesis [19], this is the first indication that p190B acts as a mitotic regulatory protein. P190B and p190A appear to have unique localization patterns and functions during mitosis. The absence of p190B from the cleavage furrow, the site of p190A action [19], and its presence at the centrosomes, is consistent with p190B and p190A playing different roles in mitosis. Our data demonstrate that p190B affects chromosome segregation, whereas p190A has been reported to regulate cytokinesis [19,20,21,22]. Furthermore, p190B deficiency did not affect the formation of multinucleated cells, which result from failed cytokinesis, suggesting that it is dispensable for cytokinesis. Taken together, these data demonstrate a novel role for p190B in mitosis and indicate that p190B and p190A have distinct functions during mitosis and cytokinesis.

P190A protein levels decrease during mitosis, and proteosome-mediated degradation of p190A is required for completion of cytokinesis [19,22]. Our data indicate that while total p190B protein levels do not change, p190B is differentially tyrosine phosphorylated during mitosis. Furthermore, data from a screen of mitotic phosphorylated peptides [25] showed that p190B is Ser/Thr phosphorylated during mitosis. Phosphorylation is a mechanism that is known to play an important role in regulating protein translocation and activity during mitosis [25], and we hypothesize that Tyr and Ser/Thr phosphorylation may control the localization and activity of p190B during different mitotic events. Future studies will be needed to further characterize p190B phosphorylation states and their roles in spindle function.

Given the defects in MT-kinetochore attachment caused by loss of p190B expression, we hypothesized that activity of the chromosome passenger complex (CPC) that regulates MT-kinetochore attachment at metaphase might be impaired [36]. However, the localization of two key CPC proteins, Survivin and Aurora B, was not altered in p190B deficient cells suggesting that CPC function is not affected by p190B loss. Microtubule attachments to kinetochores during mitosis are highly dynamic [37], and Rho GTPases are important regulators of microtubules dynamics [32]. We therefore considered the possibility that deregulation of Rac, a direct target of p190B [16], might perturb microtubule-kinetochore attachments and chromosome segregation in p190B deficient cells. Treatment of mitotic cells with the Rac inhibitor NSC 23766 reduced the incidence of lagging chromosomes to control levels, suggesting that altered Rac activity in the p190B deficient cells enhances chromosome segregation defects in MCF-7 and Hela cells.

Recent studies investigating aneuploidy as a potential cause of cancer have revealed that large-scale aneuploidy causes mitotic catastrophe and has a tumor suppressor function, while small-scale aneuploidy allows daughter cells to evade cell death and may promote tumorigenesis [38]. While we cannot directly compare the effects of altered p190B expression in MCF-7 and Hela cells to the stochastic process of tumor formation *in vivo*, we hypothesize that p190B may affect mammary tumorigenesis by disrupting mitosis and promoting varying degrees of aneuploidy. Future studies will be required to determine the contribution of p190B’s mitotic functions to tumor formation and progression.

## 4. Experimental Section

### 4.1. Cell Culture

MCF-7 human breast cancer cells stably expressing the rtTA transgene [39] and Hela cells (ATCC) were used for all *in vitro* experiments. MCF-7 cells were cultured in growth medium (DMEM high-glucose, 10% Tet-System FBS, 1% insulin, 1% sodium pyruvate, with or without geneticin antibiotic) in 5% CO_2_ at 37 °C. Hela cells were cultured in growth medium (DMEM high-glucose, 10% FBS, with or without gentamicin). Assays were performed in 8-well chamber slides, glass coverslips, or culture dishes (BD Biosciences, Franklin Lakes, NJ, USA).

### 4.2. Transfection and Synchronization

For knockdown experiments, cells were seeded at 10,000 cells per well in an eight-well chamber slide or 500,000 cells per 60 mm plate in antibiotic-free growth medium at day 1 and allowed to grow for 18–24 h at 5% CO_2_ at 37 °C. Cells were transfected on day 2 with Oligofectamine transfection reagent (Invitrogen, Camarillo, CA, USA) and 100 nM final concentration of siRNA against p190B, or a scrambled control siRNA. Two siRNAs (Dharmacon, Chicago, IL, USA) against p190B were used (#1: GCUGAUACAACCACAAUUA and #2: GGAAUCAGUUAAACACAAU. For some experiments, cells were arrested at prometaphase by treatment with 40 ng/mL nocodazole (Sigma, St. Louis, MO) 24–30 h after siRNA transfection. Arrested cells were then released by addition of fresh growth medium the following day (48 h after transfection) and incubated for 30–90 min to enrich for cells in metaphase and anaphase. The zero time point cells were not washed with fresh medium prior to fixation. Asynchronous cells were not treated with nocodazole.

Transfection of GFP tagged p190B plasmid (Origene, Rockville, MD, USA) into MCF-7 cells for immunostaining and localization was done using 0.5–1 μg of plasmid and 1.5 μL Fugene 6 transfection reagent (Roche) per well of 12-well culture plate. Cells were plated at 100,000 per well, grown overnight, and then transfected with plasmid. Cells were fixed and stained 48 h after transfection.

### 4.3. Antibodies

The following antibodies were used for immunofluorescence staining: α-tubulin (1:500, Abcam, Cambridge, MA, USA), HEC1 (1:500, clone 9G3.23, Novus Biologicals, Littleton, CO, USA), pericentrin (1:200, Abcam), Survivin (1:50, Santa Cruz; Biotechnology, Inc., Santa Cruz, CA, USA), Aurora B (1:1000, BD Transduction Laboratories, San Jose, CA, USA), and CREST serum (1:1000, Immunovision, Springdale, AR, USA). The following antibodies were used to probe Western blots: p190B (1:250, BD Transduction Laboratories), p190A (1:1000, BD Transduction Laboratories), β-actin (1:2000, Sigma, St Lois, MO, USA). For immunoprecipitation of tyrosine-phosphorylated proteins, p-Tyr antibody (BD Transduction Laboratories) was used.

### 4.4. Immunoprecipitation

Asynchronous and nocodazole synchronized MCF-7 cells were lysed with RIPA buffer (150 mM NaCl, 0.1% SDS, 0.5% sodium deoxycholate, 1% Triton X-100, and 10 mM Tris, pH 7.5), and 300 μg of protein were incubated with 4 μg p-Tyr antibodies rocking overnight at 4 °C. The lysate and antibodies were subsequently incubated with protein A/G beads (Thermo Scientific, Rockford, IL, USA) rocking at room temperature for 2 h. Beads were washed several times in RIPA buffer to remove unbound proteins. Beads were boiled in denaturing SDS-PAGE loading dye buffer for 10 min and centrifuged. The supernatant was used for subsequent SDS-PAGE and Western blotting.

### 4.5. Preparation of Cell Lysates and Western Blotting

Cells were lysed using RIPA buffer with protease inhibitor cocktail (Roche or Thermo Scientific) and phosphatase inhibitors (sodium fluoride and sodium orthovanadate) on ice for 10 min, and lysates were cleared by centrifugation and immediately frozen in aliquots. Protein concentrations were determined using the BCA Assay (Thermo Scientific). Proteins were separated by SDS-PAGE and transferred to PVDF membrane (Millipore, Bedford, MA, USA). Membranes were blocked in 5% milk in Tris-buffered saline with 0.05% Tween 20 (TBST) followed by incubation with antibodies overnight at 4 °C in blocking buffer or 3% BSA/TBST solution. Secondary HRP-conjugated antibody was applied for 45–60 min in milk/TBST. Blots were incubated with chemiluminescence reagents (Thermo Scientific or GE) and imaged using a Kodak Gel Logic 1500 system or by exposure to radiological film. Relative protein expression was quantified by densitometry using ImageJ software, and protein expression was normalized to β-actin levels.

### 4.6. Immunofluorescence Staining

Cells were extracted for 10 min at 37 °C in extraction buffer (100 mM PIPES, 1 mM MgCl_2_, 1 mM CaCl_2_, 0.5% Triton X-100, pH 6.8), fixed for 20 min at 37 °C in freshly prepared 2–4% paraformaldehyde in PBS, permeabilized for 10 min at room temperature in 0.5% Triton X-100 in PBS, washed 3 times for 5 min each in glycine wash buffer (130 mM NaCl, 7 mM Na_2_HPO_4_, 3.5 mM NaH_2_PO_4_, and 100 mM glycine), blocked for 30 min in IF buffer (130 mM NaCl, 7 mM Na_2_HPO_4_, 3.5 mM NaH_2_PO_4_, 7.7 mM NaN_3_, 0.1% BSA, 0.2% Triton X-100, and 0.05% Tween-20) plus 10% goat serum, incubated with primary antibodies in blocking buffer for 1 h at room temperature, washed three times for 5 min each in IF buffer, incubated with secondary antibodies conjugated with Alexa 555 and Alexa 488 diluted 1:1000 in blocking buffer for 1 h, washed 3 times in IF buffer, incubated with To-Pro-3 (Invitrogen) diluted 1:200 in PBS for 15 min, washed with PBS, and mounted with Vectashield mounting medium with DAPI (Vector Laboratories, Burlingame, CA, USA). Images were captured using a Zeiss LSM710 scanning confocal microscope with Zen imaging software. For fluorescence intensity quantification of Survivin, Aurora B, and CREST, images of metaphase cells were captured using equivalent zoom and gain settings and then analyzed for intensity using Zen software. Intensity values for Survivin and Aurora B were normalized to the CREST intensity value of the same cell.

### 4.7. Inhibitor Experiments

Rac inhibitor experiments were performed using the siRNA transfection protocol outlined above. Rac inhibitor, NSC 23766 (Tocris Bioscience, Minneapolis, MN, USA) was added to a final concentration of 25 μM to nocodazole-synchronized MCF-7 or Hela cells at the time of release from nocodazole. Cells were fixed and stained according to the above protocol after 70 min.

### 4.8. Flow Cytometry

MCF-7 cells were plated and transfected according to above siRNA protocol and grown in 60 mm plates for 48 h after transfection. Cells were trypsinized briefly with 0.25% Trypsin-EDTA (Invitrogen), pelleted by centrifugation at 200 g for 5 min, resuspended in PBS, fixed by the gradual addition of 100% ethanol while vortexing, incubated on ice for 15 min, centrifuged at 453 g for 5 min, and resuspended in staining solution (0.05 mg/mL propidium iodide, 0.1 mg/mL RNaseA, 0.05% Triton X-100 in PBS). Stained cells were mixed twice with a 26 gauge needle and syringe and passed through a 40 μm filter. Analysis was performed on a Beckman-Coulter FC 500 series flow cytometer. Triplicate samples were analyzed and the experiment was repeated twice.

### 4.9. Statistical Analysis

A two-tailed, paired t-test was used to compare experiments performed in parallel. All experiments data are representative of at least three independent experiments. For analysis of chromosome segregation defects a minimum of 100 cells were analyzed per experiment. A *p*-value of <0.05 was considered statistically significant. 

## 5. Conclusions

These studies are the first to implicate p190B RhoGAP as a regulator of mitosis. Future studies will be required to evaluate the contribution of p190B’s mitotic functions to normal and neoplastic development *in vivo*.
